# Familial florid cemento-osseous dysplasia in two young sisters: case report and literature review

**DOI:** 10.1038/s41415-026-9952-8

**Published:** 2026-09-25

**Authors:** Luiz Pedro Mendes de Azevedo, Isis Raquel Ghelardi, Verônica Caroline Brito Reia, Denise Tostes Oliveira, Paulo Sérgio da Silva Santos

**Affiliations:** Department of Surgery, Stomatology, Pathology and Radiology, Bauru School of Dentistry, University of São Paulo, Brazil

## Abstract

Recently identified as a variant of cemento-osseous dysplasia, familial florid cemento-osseous dysplasia (FFCOD) differs from the sporadic florid variant in both clinical and radiographic behaviour and has an autosomal dominant inheritance pattern. The aim of this study is to describe the clinical, imaging, and histopathological findings of two white young sisters who were diagnosed with FFCOD, as well as the difficulties in diagnosing and treating this condition. Multifocal fibro-osseous lesions of the jaws with early onset and maturation, and association with multiple unerupted teeth were found in both patients. Other fibro-osseous lesions and systemic disorders, such as fibrous dysplasia, familial gigantiform cementoma, gnathodiaphyseal dysplasia, Paget's disease of bone, and hyperparathyroidism-jaw tumour syndrome, were taken into consideration and ruled out during the diagnostic procedure. Even though conservative management is the preferred course of treatment for FFCOD, the frequent association with unerupted teeth presents an additional challenge for the management of lesions and oral rehabilitation of these patients. Therefore, more research is needed to better understand this variant and develop safer therapeutic and rehabilitative strategies.

**Supplementary Information:**

Zusatzmaterial online: Zu diesem Beitrag sind unter https://doi.org/10.1038/s41415-026-9952-8 für autorisierte Leser zusätzliche Dateien abrufbar.

## Key points

- Familial florid cemento-osseous dysplasia (FFCOD) may affect younger, white patients and exhibit early, rapidly maturing multifocal jaw lesions.
- Recognition of clinical, imaging, and histopathological correlations is essential to distinguish FFCOD from other fibro-osseous and metabolic bone disorders.
- Conservative management is recommended; however, frequent association with unerupted teeth raise questions about orthodontic treatment and oral rehabilitation.

**Supplementary Information:**

Zusatzmaterial online: Zu diesem Beitrag sind unter https://doi.org/10.1038/s41415-026-9952-8 für autorisierte Leser zusätzliche Dateien abrufbar.

## Introduction

Cemento-osseous dysplasia (COD) is a benign fibro-osseous lesion characterised by replacement of normal bone with dysplastic fibrous connective tissue intermixed with immature bony trabeculae and cementum-like mineralised material.^1^ It may affect the apical region of vital anterior teeth (periapical variant), present as a solitary lesion typically confined to the posterior mandible (focal variant), or involve multiple quadrants (florid variant), the latter being more common in middle-aged Black women.^2^ Radiographically, these lesions present distinct features depending on the stage of development, appearing initially radiolucent and progressively becoming mixed and radiopaque.^1^^,^^2^^,^^3^

The fifth edition of the World Health Organization (WHO) *Classification of head and neck tumours* introduced a fourth variant of COD, designated familial florid cemento-osseous dysplasia (FFCOD), which has a hereditary component and presents clinical and radiographic features distinct from those of the ‘sporadic' florid variant.^4^^,^^5^

FFCOD may affect younger individuals, shows a less marked predilection for females, and, due to its autosomal dominant inheritance pattern, is also frequent in white patients. The lesions may cause pronounced bone expansion and, as they develop rapidly from the fibrous (radiolucent) phase to the sclerotic (radiopaque) phase, mainly in young patients, they are often associated with impacted teeth.^4^ The condition may evolve with significant clinical manifestations, such as bone expansion, pain, tooth mobility, and an increased risk for the development of osteomyelitis, which is the primary reason why biopsies and/or surgical intervention are contraindicated.^6^^,^^7^

The differential diagnosis is complex and requires the integration of clinical, radiographic, and histopathological data, making it essential to distinguish this condition from other fibro-osseous lesions, as well as from chronic sclerosing osteomyelitis and Paget's disease of bone.^5^^,^^8^^,^^9^ In view of these aspects and the complexity of the diagnostic process, this study aims to report the cases of two young sisters diagnosed with FFCOD, based on the CARE guideline,^10^ highlighting the clinical, radiographic, and histopathological features, as well as the diagnostic and therapeutic challenges involved.

## Case 1

**Fig. 1:**
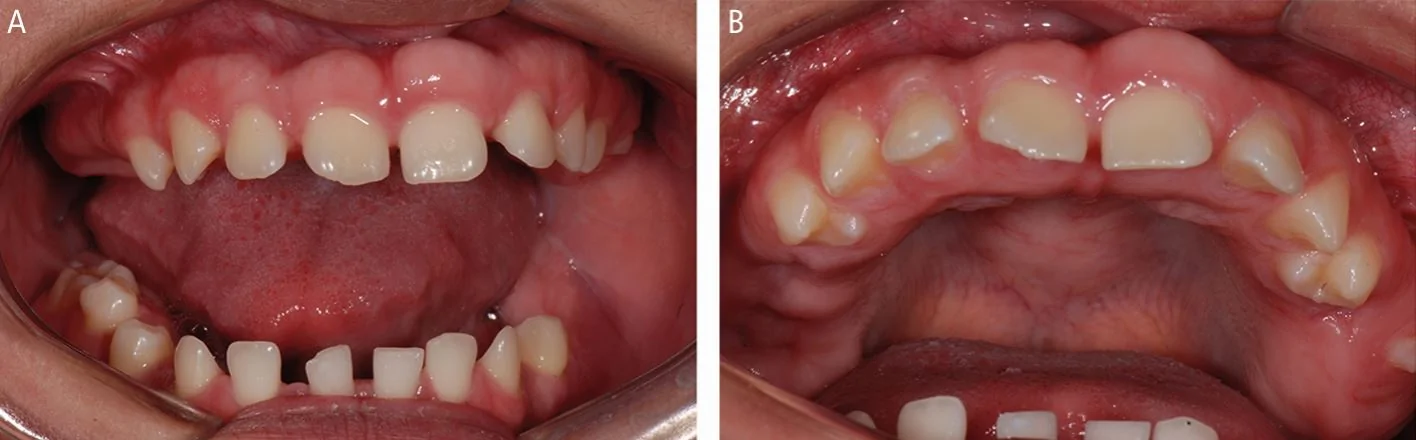
Case 1 (at 18 years old), presenting with multiple diastemas, absence of mandibular (A) and maxillary molars (B), and buccolingual enlargement of both maxillary and mandibular alveolar ridges (A, B)

A white 18-year-old woman was referred for evaluation of an intraosseous lesion. The patient reported that, at ten years of age, she was taken for dental evaluation due to delayed tooth eruption and pain. The dentist extracted her deciduous mandibular molars and noticed mandibular lesions on radiograph examination, which were followed for eight years. The medical and dental history included nervous gastritis, use of oral contraceptives, diabetes in relatives (maternal grandparents and uncles/aunts), and haemorrhage during dental extractions. There was no history of bone fractures. On general and extra-oral physical examination, the patient was normotensive and presented with short stature, slight limb shortening, hypertelorism, prominence of the upper third and hypoplasia of the middle third of the face, upslanting palpebral fissures, small ears with folded helices, and widening of the alar bases. Intra-oral examination revealed exostoses in the anterior maxilla, buccolingual enlargement of both maxillary and mandibular alveolar ridges in the molar regions, and generalised diastemas (Fig. 1).

**Fig. 2:**
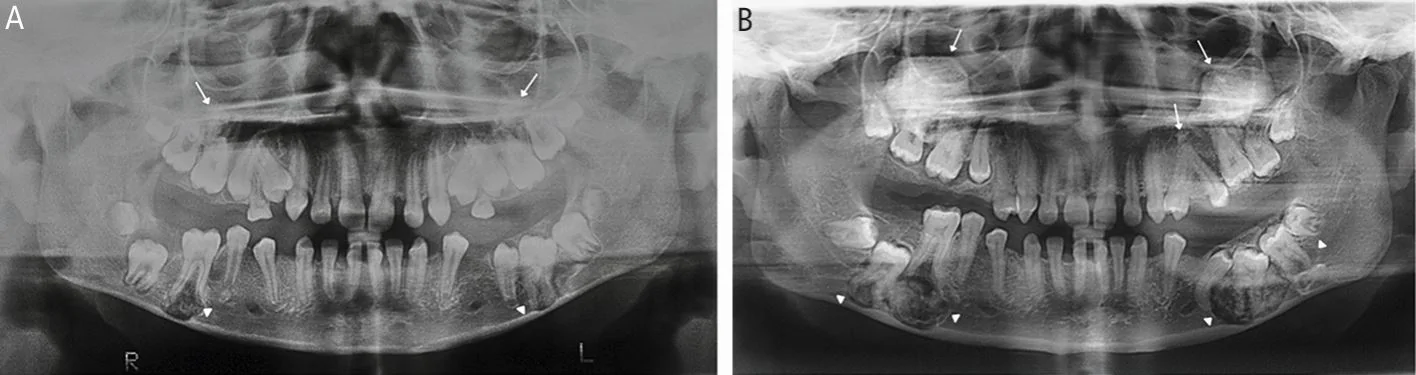
Panoramic radiograph of Case 1. (A) At 12 years of age, radiopaque lesions over the apical regions of the maxillary molars (arrows), and mixed radiopaque and radiolucent lesions between the root apices of the mandibular molars (arrowheads). (B) At 18 years of age, progression in size of the lesions associated with multiple unerupted teeth; the maxillary lesions appear completely radiopaque, with possible extension into the maxillary sinuses (arrows), whereas the mandibular lesions exhibit a mixed-density appearance (arrowheads)

On panoramic radiograph, unerupted teeth 18, 17, 16, 15, 28, 27, 26, 38, 37, 36, 35, 48 and 47 were observed. Teeth 24 and 25 were mesially and distally angulated, respectively. Well-defined radiopaque images were projected over the apical regions of the maxillary molars and the maxillary sinuses bilaterally. In addition, bilateral mixed radiolucent and radiopaque images, well-defined and surrounded by a radiolucent halo, were observed in the apical region of teeth 35–37 and 46–48 (Fig. 2B). The patient also presented a previous panoramic radiograph, obtained seven years earlier, which showed initial bilateral radiolucent mandibular lesions already exhibiting a mixed-density pattern, as well as bilateral radiopaque lesions in the maxilla (Fig. 2A). Considering the clinical and radiographic findings, the diagnostic hypotheses included florid cemento-osseous dysplasia (FCOD) and Paget's disease of bone. The adopted management consisted of periodic follow-up and referral to a medical geneticist, who found no evidence of extragnathic lesions. No genetic testing was performed.

**Fig. 3:**
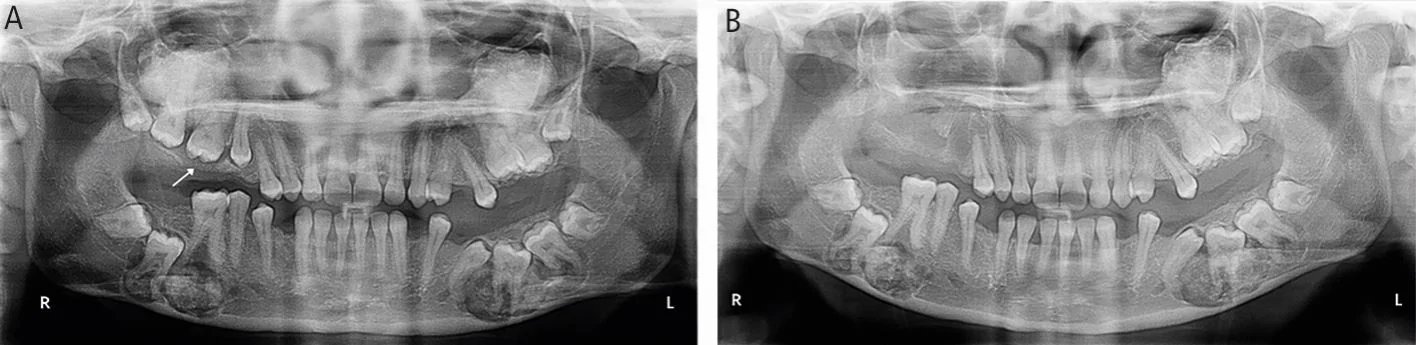
Panoramic radiographs of Case 1 (at 20 years old). (A) Focal bony discontinuity in the region of the crown of tooth 16 (arrow). (B) Radiographic appearance 22 days after surgical removal of the lesion in the right maxilla and extraction of 15, 16, 17, and 18

The patient did not attend the scheduled follow-up appointments as instructed and returned only two years later. At that time, she was undergoing orthodontic treatment but reported recurrent infectious episodes with suppuration in the right maxilla over the previous eight months, which had been managed at an urgent dental care service. Radiographic imaging demonstrated an increase in the extent of the lesion compared with previous examination, and discontinuity of the bone crypt of tooth 16 was observed, suggesting communication between this area and the oral cavity (Fig. 3A). Given the history of recurrent infection, the right maxillary region was surgically approached, with extraction of teeth 15, 16, 17, and 18, followed by enucleation and curettage of the dysplastic lesion, which extended into the maxillary sinus. The procedure was completed with a buccal flap and sutures. The patient remained asymptomatic during the post-operative period (Fig. 3A).

**Fig. 4:**
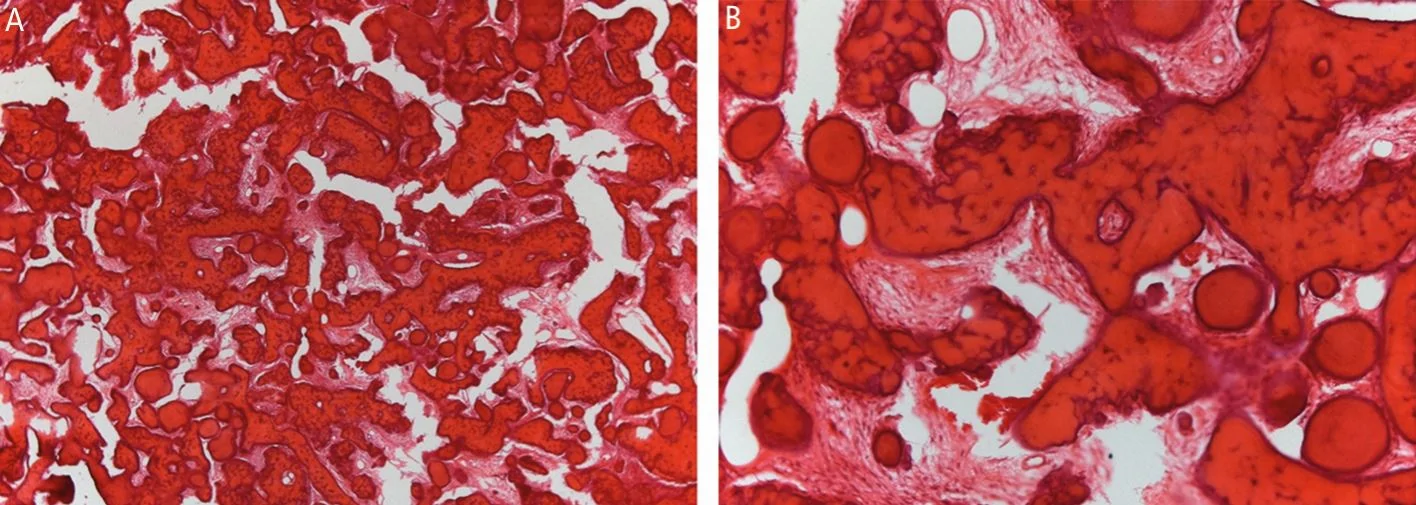
(A, B) Photomicrographs of Case 1 showing bone and cementum-like mineralised tissues permeated by fibrous connective tissue (H+E. Original magnification – A: 50x; B: 400x)

The specimen consisted of irregular fragments of hard tissue with a firm consistency and brownish colour, measuring 5.5 × 3.5 × 1.5 cm, accompanied by two intact premolars and two molars, as well as hard tissue adherent to the tooth surfaces of the molars and a fragment of soft tissue with elastic consistency and white colour, measuring 2.5 × 1.2 × 0.6 cm. Histopathological examination of haematoxylin and eosin (H+E)-stained sections showed proliferation of spindle cells arranged randomly or in coarse fascicles permeating numerous spheroidal deposits of cementum-like mineralised tissue, forming an expansive mass (Fig. 4). There was also cellular fibrous connective tissue with spindle cells arranged in fascicles permeated by small islands of odontogenic epithelium. The surfaces of the examined fragments showed stratified squamous lining epithelium, variably hyperplastic or atrophic. In some areas, the lining epithelium corresponded to reduced enamel epithelium. A severe mononuclear inflammatory infiltrate, composed of lymphocytes and plasma cells and rich in Russell bodies, was also observed, indicating persistent antigenic stimulation, findings consistent with chronic pericoronaritis.

The clinical and radiographic features, associated with the histopathological findings supported the diagnosis of ‘sporadic' FCOD at that moment.

**Fig. 5:**
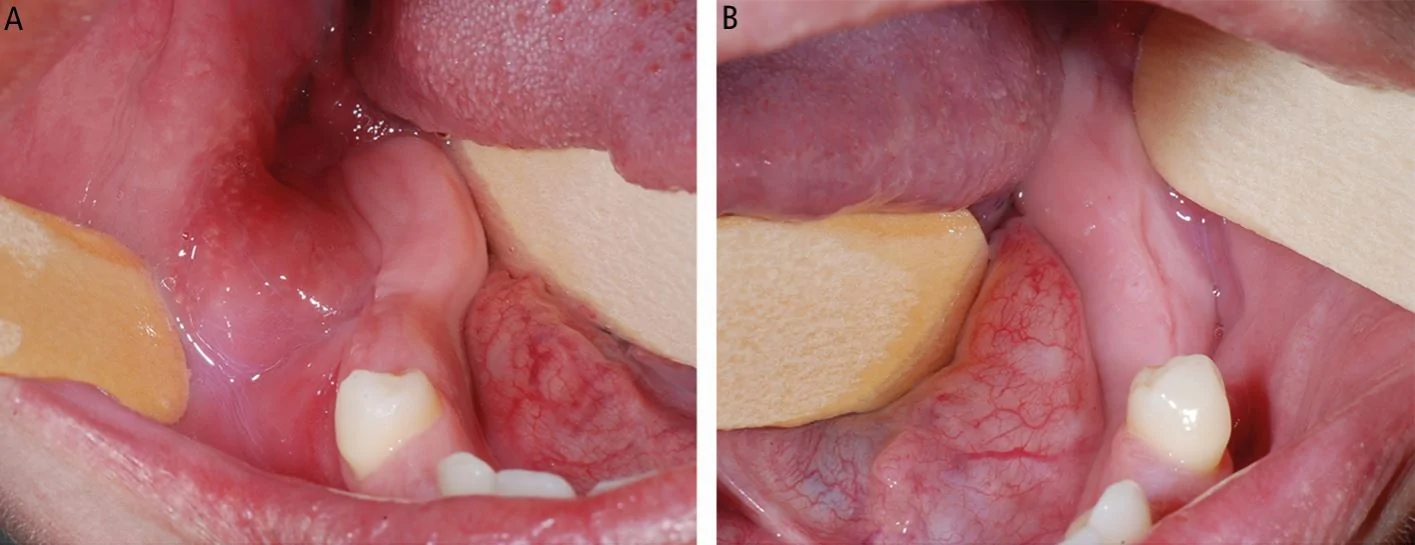
(A, B) Case 1 (at 29 years old) presenting with bilateral mandibular swelling, with obliteration of the vestibular sulcus on the right side

**Fig. 6:**
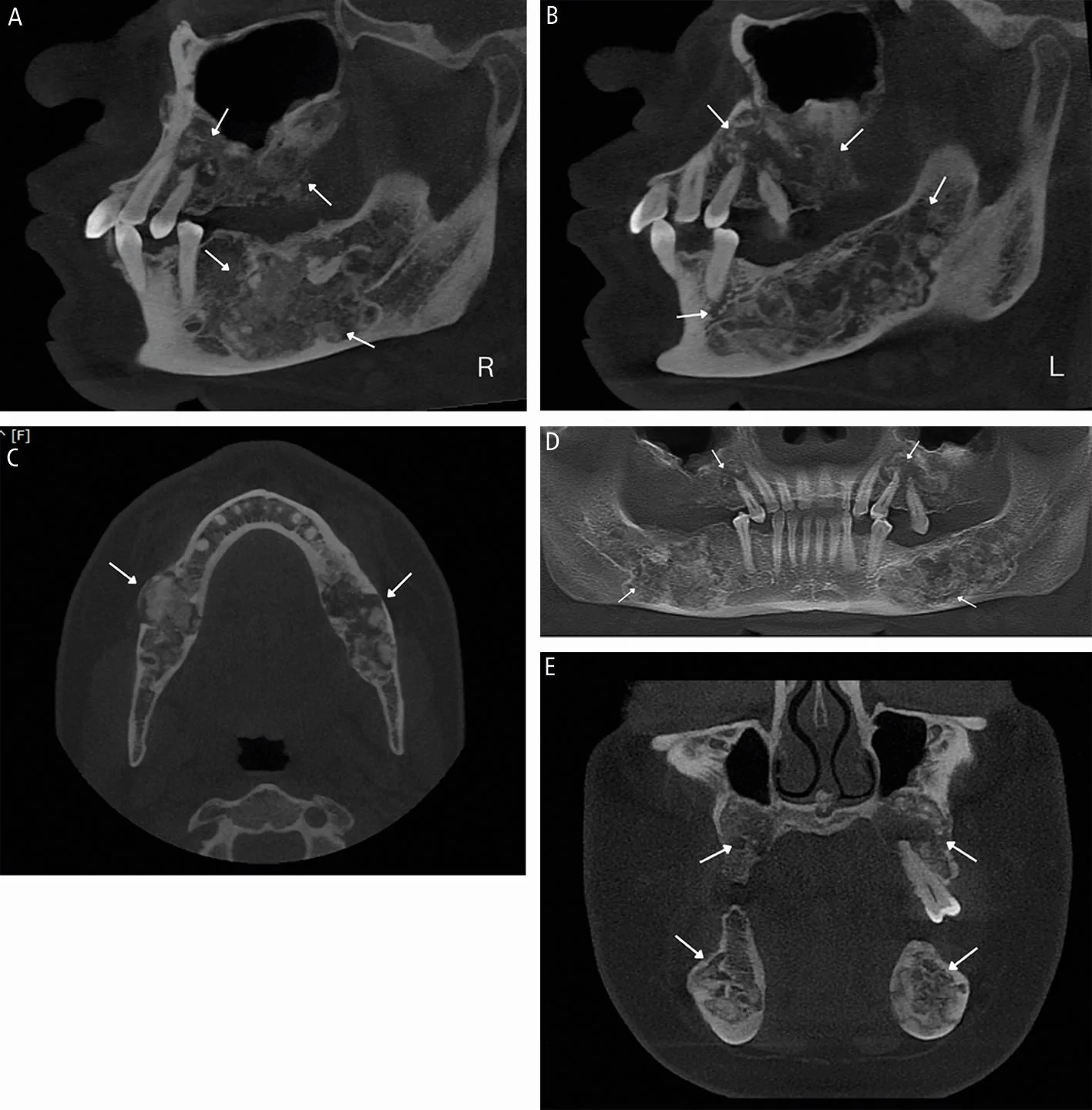
Sagittal (A, B), axial (C), panoramic (D), and coronal (E) CBCT reconstructions of Case 1 at 29 years of age, demonstrating mixed-density lesions in all quadrants (arrows), with buccolingual bone expansion in both the maxilla and mandible

After the surgical management and the initial postoperative visits, the patient discontinued follow-up and returned nine years later. At that time, she reported that the remaining quadrants had been surgically approached for removal of the lesions and the involved teeth. She was unable to clarify the reason for this intervention but reported paraesthesia of the right lower lip. Intraoral examination revealed enlargement of the right mandibular region with obliteration of the vestibular sulcus (Fig. 5). Cone beam computed tomography (CBCT) showed mixed radiolucent and radiopaque lesions associated with buccolingual bone expansion in all four quadrants (Fig. 6). Based on these findings, the diagnostic hypotheses included recurrent FCOD with paraesthesia associated with active disease and/or secondary to the previous surgical intervention. Laboratory tests confirmed normal serum levels of alkaline phosphatase, osteocalcin, triiodothyronine, thyroxine, thyroid-stimulating hormone, and parathyroid hormone. The patient remains under clinical and imaging follow-up, with no progression of the lesions to date.

## Case 2

**Fig. 7:**
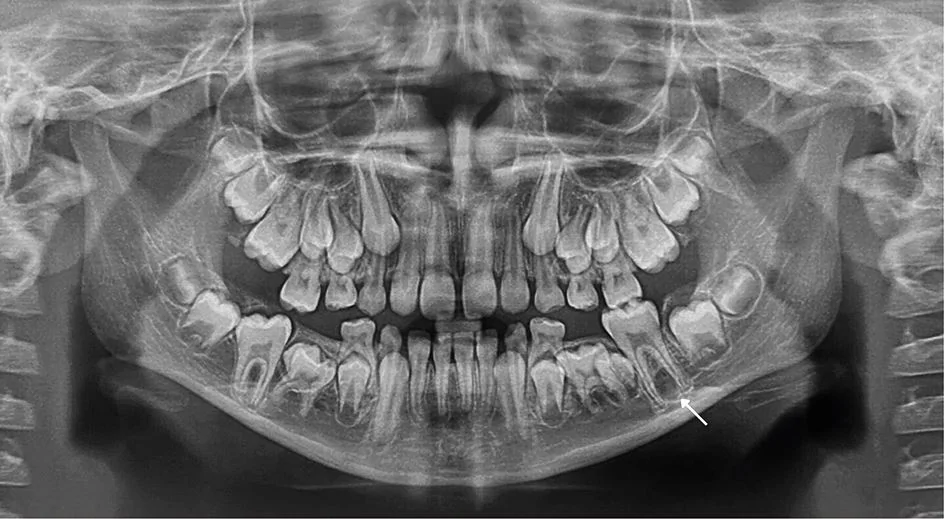
Panoramic radiograph of Case 2 (at ten years old) previously obtained, which shows a radiolucency in the interradicular region of tooth 36 (arrow)

A 13-year-old white girl, the sister of the patient in Case 1, was referred for evaluation two years after the referral of Case 1 due to the presence of an intraosseous lesion observed on panoramic radiograph since ten years of age, located in the interradicular region of the sound tooth 36 (Fig. 7).

**Fig. 8:**
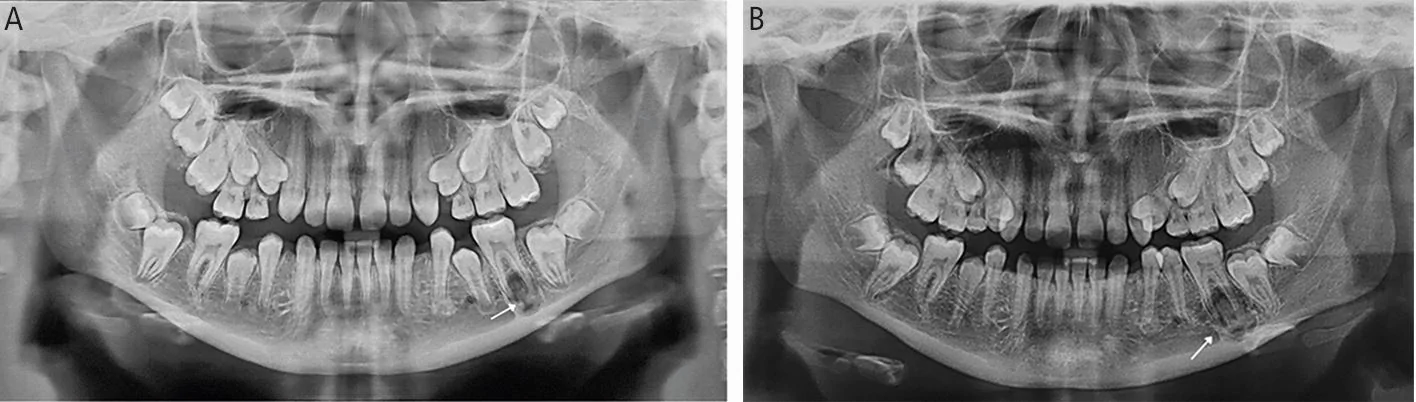
Panoramic radiographs of Case 2 at 13 years (A) and 15 years (B), demonstrating progression of the mixed-density lesion between the roots of tooth 36 (arrows), multiple unerupted permanent teeth, and prolonged retention of primary teeth

The physical examination revealed no abnormalities. A panoramic radiograph obtained at the time of the appointment showed that the lesion had increased in size and exhibited a mixed-density pattern. Additional findings included: unerupted teeth 18, 17, 15, 14, 24, 25, 27, 28, 38, 37, 47, and 48; incomplete root formation and prolonged retention of primary teeth 55, 54, 64, and 65; and teeth 16, 35, and 45 likely in course of eruption (Fig. 8A). The initial diagnosis hypothesis was focal COD, since the radiographic alterations were confined to the region of tooth 36. At that time, periodic follow-up was adopted. Subsequently, after radiographic evidence of multiple quadrants involved, the diagnosis hypothesis was revised to FCOD (Fig. 8B).

**Fig. 9:**
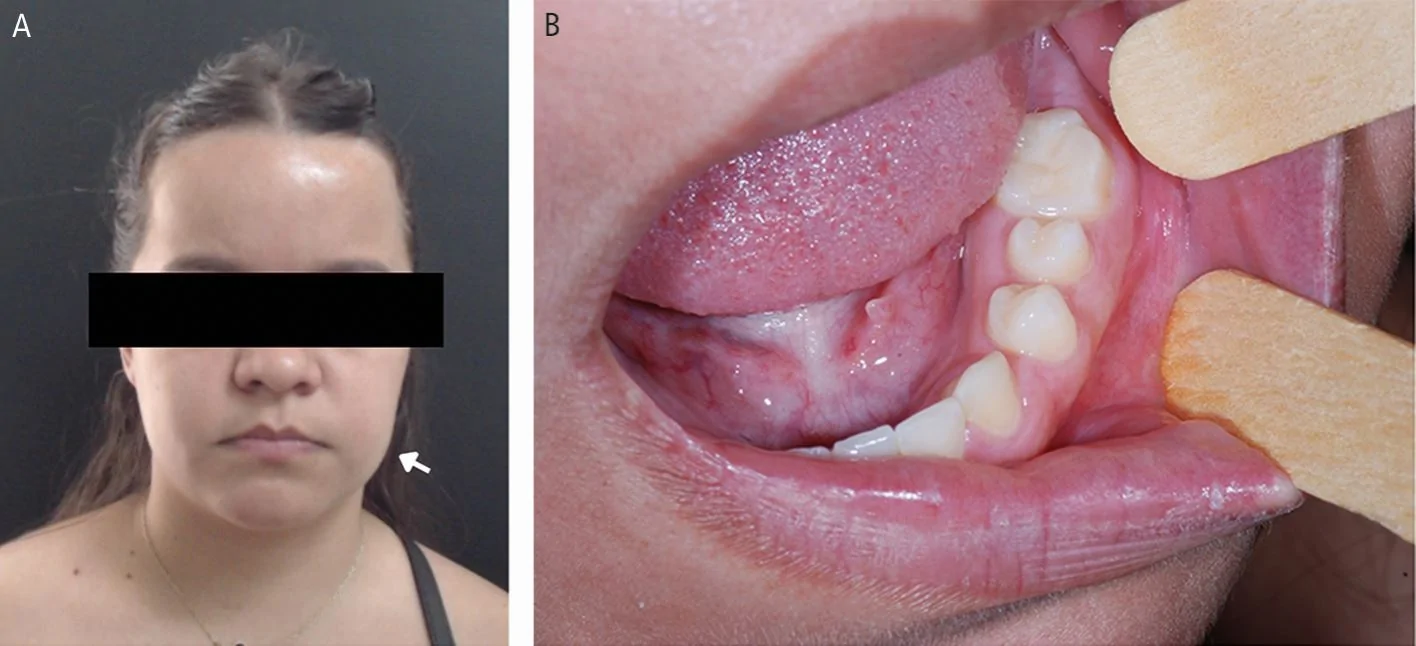
Clinical images of Case 2 at 23 years of age. (A) Facial asymmetry due to swelling in the left mandibular region (arrow). (B) Buccolingual swelling of the left mandible in the region of teeth 35 and 36

**Fig. 10:**
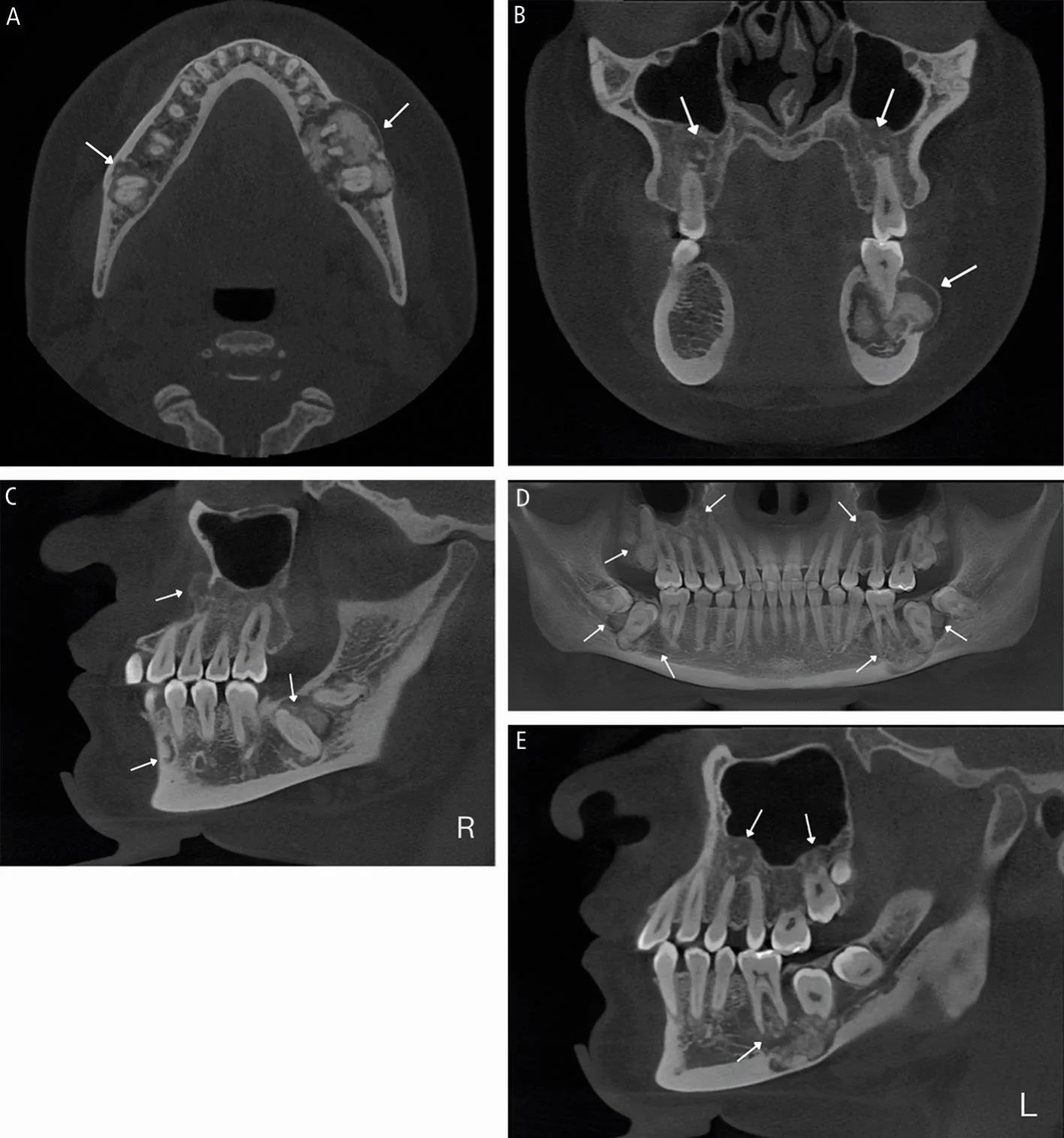
Axial (A), coronal (B), sagittal (C and E), and panoramic (D) CBCT reconstructions of Case 2 at 23 years of age, demonstrating mixed-density lesions (arrows) and unerupted teeth in all four quadrants, with bone expansion in the maxilla bilaterally and in the left mandible

The patient discontinued dental follow-up for ten years and returned reporting spontaneous facial pain. She reported having undergone orthodontic treatment at another service, which was later interrupted. Extra-oral examination revealed a firm swelling in the left mandibular region, also evident intra-orally in the region of teeth 35 and 36, in addition to multiple missing maxillary and mandibular teeth (Fig. 9). CBCT showed unerupted teeth 18, 17, 27, 28, 38, 37, 47, and 48; and well-defined lesions with a mixed-density appearance projected in the premolar and molar region of all four quadrants (Fig. 10). Considering the clinical and radiographic findings, together with patient's family history and the absence of a history of bone fractures, a final diagnosis of FFCOD was established for both cases. The instituted management consisted of clinical and radiographic follow-up.

## Discussion

FFCOD belongs to the group of benign fibro-osseous lesions, which share histological features and for which correlation of clinical and imaging findings is mandatory for diagnosis.^11^^,^^12^ Among the main conditions included in the differential diagnosis of FFCOD are fibrous dysplasia, familial gigantiform cementoma (FGC), gnathodiaphyseal dysplasia (GDD), Paget's disease of bone and hyperparathyroidism-jaw tumour syndrome (HPT-JT).^5^^,^^13^

Fibrous dysplasia, although it may involve both the maxilla and mandible concomitantly and affect young patients in the second and third decades of life, typically presents radiographically with a classic ground-glass opacification with poorly defined borders,^14^ which differs from the cases described here. FGC, a lesion previously considered a variant of COD due to clinical, radiographic, and histopathological similarities, also demonstrates early onset; however, it is characterised by progressive and marked bone expansion, leading to significant facial deformity before lesion maturation.^5^^,^^15^ Some authors report no extragnathic involvement in this condition,^13^ whereas others describe long bone fractures and elevated serum alkaline phosphatase levels,^15^^,^^16^ findings not identified in our cases.

GDD, in turn, is a rare genetic disorder in which patients present with fibro-osseous lesions of the jaws, osteopenia, bone fragility, bowing, and cortical thickening of the long bones,^15^^,^^17^^,^^18^ which were absent in the reported cases. Paget's disease of bone, although reported in the literature as a differential diagnosis of FFCOD, exhibits features distinct from those observed in the two cases presented. It is a metabolic condition characterised by disorganised bone remodelling and abnormal bone deposition, resulting in bone weakening, pain, deformities, and pathological fractures, and is uncommon in patients younger than 55 years. Lesions in the intermediate stage of maturation may present a ground-glass pattern, later evolving to a cotton-wool pattern, and may involve extragnathic bones.^19^

HPT-JT is an autosomal dominant condition caused by a mutation in the HRPT2 gene, which encodes parafibromin, and is most commonly diagnosed between the second and fourth decades of life.^20^^,^^21^ Clinically, it is characterised by hyperparathyroidism, multiple parathyroid adenomas, renal and uterine tumours, and multiple ossifying fibromas in the jaws.^5^^,^^20^ The absence of these features allowed this hypothesis to be excluded.

Other jaw lesions may resemble FFCOD, depending on their stage of development. These include ossifying fibroma, classified as a neoplasm with continuous expansive growth;^5^ chronic diffuse sclerosing osteomyelitis, which may be asymptomatic but is generally associated with odontogenic infection;^2^^,^^6^^,^^22^^,^^23^^,^^24^^,^^25^ cementoblastoma, a radiopaque lesion fused to the dental root;^6^ hypercementosis, which, in its early stages, may resemble FFCOD but is restricted to the dental root;^6^ and osteosarcoma, a malignant neoplasm associated with pain and rapid growth.^25^

Thus, based on the clinical, radiographic, and histopathological findings, together with the exclusion of the differential diagnosis discussed, the initial diagnosis for Case 1 was (‘sporadic') FCOD. However, some features were unusual for this condition, including its occurrence in white patients, early onset, rapid lesion maturation, and association with unerupted teeth. Years later, evaluation of Case 2 revealed similar clinical and radiographic features in the younger sister, and, together with the family history, allowed a definitive diagnosis of FFCOD, a condition recognised by WHO as a fourth variant of COD due to its behaviour distinct from the ‘sporadic' form. No additional family members were available for clinical evaluation.

A search was performed in PubMed, Scopus and Web of Science databases using the combined terms ‘familial', ‘florid', and ‘dysplasia'. Twelve studies were initially identified,^22^^,^^23^^,^^26^^,^^27^^,^^28^^,^^29^^,^^30^^,^^31^^,^^32^^,^^33^^,^^34^^,^^35^ two of which were excluded from this review,^30^^,^^33^ as the clinical findings described were consistent with conditions other than FFCOD.^5^ Overall, 37 cases have been reported in the literature, including the two presented herein. The clinical, imaging and histopathological features of the included case reports are summarised in the online Supplementary Information.

FFCOD is a condition characterised by a multifocal replacement of bone tissue in the jaws by fibrous connective tissue, immature bone trabeculae and cementum-like material. In contrast to the ‘sporadic' florid variant, which typically affects middle-aged women, FFCOD may affect younger patients.^5^^,^^12^ In the literature analysed, the diagnosis occurred before 35 years of age in 16 cases (43.2%),^22^^,^^23^^,^^27^^,^^28^^,^^29^^,^^31^^,^^32^^,^^35^ including the sisters in this study, who were diagnosed at 18 and 13 years, emphasising the early onset of this variant.

A predominance among women was observed, with 25 cases (67.6%) occurring in women (25F:12M), a proportion lower than that reported for the ‘sporadic' form, which, according to some authors, may affect women in up to 90% of cases.^36^^,^^37^ Moreover, whereas ‘sporadic' FCOD occurs predominantly in Black individuals, such predilection appears less pronounced in the familiar variant, possibly due to its autosomal dominant inheritance.^35^ In the present study, both cases occurred in white patients, as also reported by Sedano *et al*.^26^ and Musella *et al*.^27^

Regarding clinical features, most reported cases of FFCOD are associated with bone expansion,^5^^,^^22^^,^^23^^,^^26^^,^^27^^,^^29^^,^^31^^,^^32^^,^^34^ some of which asymptomatic,^26^^,^^27^^,^^28^^,^^29^^,^^31^^,^^32^^,^^34^ whereas others presented signs and symptoms of secondary infection or bone necrosis.^22^^,^^23^^,^^27^^,^^29^^,^^31^^,^^32^^,^^34^^,^^35^ In this study, Case 2 remained asymptomatic throughout follow-up. In contrast, Case 1 initially reported pain unrelated to secondary infection and later developed lower lip paraesthesia, an unusual finding described by Grun *et al*.^38^

The imaging appearance of these lesions depends on their stage of maturation. In initial stages, they exhibit a radiolucent/hypodense pattern, progressively evolving to a mixed-density appearance and ultimately to a radiopaque/hyperdense pattern.^5^ Most cases reported in the literature present a mixed-density pattern,^22^^,^^23^^,^^28^^,^^29^^,^^31^^,^^32^^,^^34^^,^^35^ which was also observed in the present cases. Additionally, in Case 1, the maxillary lesions were already radiopaque/hyperdense at 18 years of age, indicating that FFCOD lesions may progress rapidly.

FFCOD may be associated with the presence of unerupted teeth,^5^ which is believed to be related to the early maturation of lesions in dentate areas. In the present review, nine cases showed unerupted teeth,^22^^,^^23^^,^^29^^,^^34^^,^^35^ a finding also observed in the cases reported here, in which premolars and molars were involved. In contrast, the association with unerupted teeth in the ‘sporadic' variant appears to be uncommon, having been reported in only a few studies.^6^^,^^8^^,^^39^^,^^40^

Overall, the management of FFCOD is conservative, and surgical interventions, including biopsies, are contraindicated except in cases of secondary infection, due to the increased risk of osteomyelitis. The approach relies on periodic clinical and radiographic follow-up, with emphasis on preventing local factors that may lead to bone exposure or infection.^5^ In Case 1, a surgical approach in the right upper quadrant was indicated due to recurrent episodes of infection, a condition that in itself represents a risk for osteomyelitis. Notably, these episodes occurred before any surgical intervention. Radiographic imaging revealed discontinuity of the bone in the affected region, suggesting communication between the dysplastic lesion and the oral cavity, which likely facilitated contamination of the poorly vascularised tissue. Additionally, local factors such as trauma to the edentulous area, possibly from the opposing dentition during mastication, may have contributed to the development of infection. In contrast, in Case 2, conservative management proved sufficient, and no surgical approach was required during the follow-up period. While both patients underwent orthodontic treatment, only Case 1 developed infection, whereas Case 2 presented with clinical symptoms, suggesting that orthodontic forces alone were not sufficient, but might have contributed to the development of clinical manifestations.

Although FCOD is a well-established entity, the familial variant poses additional clinical challenges, as encountered in the management of both cases described, particularly with the management of multiple unerupted teeth. On the one hand, surgical interventions are not recommended; on the other, malocclusions resulting from tooth non-eruption raise questions about the feasibility of orthodontic and rehabilitative approaches, considering the limitations imposed by bone manipulation and by the direct contact of removable prostheses with mucosa overlying dysplastic bone, which presents a major challenge for both professionals and patients. In this context, the literature still lacks specific guidelines or studies to support therapeutic and rehabilitative decision-making in such cases.

Minhas *et al*.,^25^ and Sethusa and Khan^41^ have reported the use of orthodontic treatment in patients with FCOD. Both cases involved bilateral mandibular lesions, dental malocclusion, and orthodontic management. The studies reported favourable outcomes, reinforced the importance of rigorous oral hygiene, and emphasised the contraindication of tooth extraction in these cases. However, they did not provide an in-depth evaluation of the response of dysplastic bone to orthodontic movement, nor did they propose specific treatment protocols or biomechanical considerations for such cases. In the cases reported in this study, despite the hypothesis that orthodontic treatment may have contributed to the onset of symptoms, the absence of objective diagnostic methods to assess bone response does not allow the establishment of a causal relationship between orthodontic forces and clinical manifestations. Other contributing factors, such as local trauma, microbial contamination, and individual susceptibility, should also be considered.

From a therapeutic perspective, the presence of multiple unerupted teeth significantly limits conventional orthodontic and rehabilitative approaches. Management should focus on careful monitoring, maintenance of periodontal health, and avoidance of invasive procedures. When orthodontic treatment is considered, the lack of evidence regarding the response of dysplastic bone to orthodontic movement represents an additional challenge, as no specific protocols or guidelines have been established. In terms of oral rehabilitation, alternatives that minimise trauma to the mucosa overlying dysplastic bone should be considered. The use of resilient lining materials in removable prostheses may reduce mechanical trauma and the risk of mucosal breakdown, particularly in edentulous areas overlying affected bone, and should be further investigated as a potential strategy to facilitate rehabilitation in these cases.

Thus, further studies are needed to clarify the biological behaviour and specific characteristics of FFCOD, as well as the response of dysplastic bone to orthodontic movement, and to establish safe and effective rehabilitation strategies for affected patients.

## Conclusion

FFCOD presents clinical features that differ from those of the ‘sporadic' florid variant, affecting younger and white patients, and often exhibiting early lesion maturation. The cases involving the two sisters reported in this study highlight these particularities, illustrating the clinical, radiographic, and histopathological aspects of this condition, as well as the diagnostic and therapeutic challenges involved in its management.

Although a conservative approach is recommended for FFCOD, its frequent association with unerupted teeth represents a challenge for lesion management and oral rehabilitation, underscoring the need for further studies to improve understanding of this variant.

## Supplementary Information

online Supplementary Information (PDF 120KB)
