# Supplementary material for: Familial florid cemento-osseous dysplasia in two young sisters: case report and literature review
Source: Br Dent J. 2026 Sep 25;241(6):395–401. doi: 10.1038/s41415-026-9952-8 (PMC13614892; doi:10.1038/s41415-026-9952-8)
Supplement: Supplementary file 1 [file 41415_2026_9952_MOESM1_ESM.pdf]

Table 1: Clinical and imaging findings in FFCOD cases reported in the literature.

Author, Country (year)	Age (years)	Sex (n)	Family history	Clinical features	Quadrant involvement	Radiographic features	Tomographic features	Histopathological features	Laboratory exam results	Management
Sedano et al., USA (1982)	40 (case 1); NR (other cases)	F = 6<br>M = 4	10 affected individuals	Asymptomatic, no infection or suppuration.	Q2, Q3, Q4	Radiopaque image; poorly defined borders; globular appearance	NR	Irregular amorphous calcification and partially calcified bone fragments	Normal levels of AP, calcium, and phosphorus (all cases)	Biopsy; clinical and radiographic follow-up
Musella et al., USA (1989)	33 (case 1)<br>71 (case 2)	F = 2<br>M = 0	2 affected individuals	Asymptomatic, without bone expansion (case 1); Bone expansions treated with surgical osteoplasty over 30 years, refractory osteomyelitis, fistula with drainage (case 2)	Q1, Q2, Q3, Q4	Radiopaque image; poorly defined borders	Cortical thickening	Predominantly sclerotic bone with minor fibrous component	Normal serum levels of AP, calcium, and phosphorus (cases 1 and 2)	Follow-up (case 1); biopsy, antibiotic therapy and hyperbaric oxygen therapy for osteomyelitis, follow-up (case 2)
Thakkar et al., UK (1993)	61 (case 1)<br>34 (case 2)<br>30 (case 3)<br>26 (case 4)	F = 3<br>M = 1	4 affected individuals	Asymptomatic	Q1, Q2, Q3, Q4	Mixed-density pattern; poorly defined borders	NR	Dense globular masses in fibrous matrix	NR	Extraction of teeth with periodontitis (case 1); follow-up (cases 1–4)
Coleman et al., South Africa (1996)	32 (case 1)<br>13 (case 2)<br>9 (case 3)	F = 2<br>M = 1	3 affected individuals	Bone expansion, infection and suppuration (case 1); Asymptomatic (cases 2-3)	Q1, Q2, Q3, Q4 (case 1)<br>Q3, Q4 (case 2 and 3)	Mixed-density pattern; poorly defined borders; unerupted teeth	NR	Cellular fibrous tissue, cementum-like masses, acellular areas, pus and debris; non-keratinized squamous epithelium	NR	Biopsy; extraction; follow-up
Hatori et al., Japan (2003)	29 (case 1)<br>62 (case 2)	F = 1<br>M = 1	2 affected individuals	Pain, pericoronaritis associated with third molar (case 1); Bone exposition, osteomyelitis, malodour (case 2)	Q1, Q2, Q3, Q4	Mixed-density pattern; poorly defined borders; lobular	Hyperdense image, hypodense halo; cortical resorption	NR	NR	Extraction; intravenous antibiotic therapy; follow-up (case 1); NR (case 2)

Srivastava et al., India (2012)	18 (case 1) NR (case 2)	F = 1 M = 1	2 affected individuals	Missing teeth, retained deciduous teeth; bone expansion, asymptomatic (case 1); Bone exposure and necrosis (case 2)	Q1, Q2, Q3, Q4	Mixed-density pattern; poorly defined borders; lobular; unerupted teeth	Hyperdense image, hypodense halo; cortical bulging	NR	Normal AP, calcium and phosphorus (case 1)	Curettage; antibiotic therapy (case 2); follow-up (cases 1 and 2)
Sim et al., South Korea (2014)	49 (case 1) 21 (case 2) 21 (case 3)	F = 3 M = 0	3 affected individuals	Asymptomatic; later pain, bone exposure, swelling after extraction of mobile teeth; rehabilitation	Q1, Q2, Q3, Q4 (case 1) Q1, Q3, Q4 (cases 2-3)	Mixed-density pattern; poorly defined borders; lobular	Hyperdense image, hypodense halo; cortical resorption and bulging	Irregular cementoid tissues and cellular fibrous stroma	Normal serum levels of AP, calcium and phosphorus (case 1-3)	Extraction of mobile teeth; curettage (case 1); biopsy; extraction; curettage; follow-up (cases 2 and 3)
Thorawat et al. Saudi Arabia and India (2015)	24 (case 1) 45 (case 2)	F = 2 M = 0	2 affected individuals	Pain, swelling, suppuration, lymphadenopathy (case 1); Asymptomatic, bone expansion (case 2)	Q1, Q2, Q3, Q4	Mixed-density pattern; poorly defined borders; lobular; unerupted teeth	NR	Irregular cementoid tissues; cellular stroma; blood vessels; chronic infiltrate	Normal levels of calcium, phosphorus, and AP (cases 1 and 2)	Biopsy; antibiotic therapy (case 1); follow-up (cases 1 and 2)
Lv et al., China (2019)	49 (case 1) NR (other cases)	F = 1 M = 3	4 affected individuals	Asymptomatic, maxillary and mandibular swelling, bone necrosis after dental extraction (case 1); Osteomyelitis (case 2); Asymptomatic (cases 3 and 4)	Q1, Q2, Q3, Q4	Irregular cloud-like radiopaque and mixed-density images (case 1)	NR	NR	Missense mutation p.C356W in anoctamin 5 (ANO5) gene	Dental extraction; excisional biopsy / surgical approach to necrotic bone (cases 1 and 2); follow-up
Nel et al., South Africa (2020)	58 (case 1) 18 (case 2) 21 (case 3)	F = 2 M = 1	3 affected individuals	Pain, facial asymmetry, swelling, bone exposure and necrosis (case 1); Asymptomatic, extraoral swelling, retained primary teeth (case 2); Bone expansion, swelling (case 3)	Q1, Q2, Q3, Q4	Mixed-density pattern; bone expansion; unerupted teeth; maxillary sinus, orbit and nasal cavity involvement	NR	Hypocellular sclerotic masses; chronic osteomyelitis; inflammatory infiltrate	NR	Surgical debridement (case 1); biopsy (cases 1 and 3); follow-up

Azevedo et al., Brazil (2026)	18 (case 1)<br>13 (case 2)	F = 2<br>M = 0	2 affected individuals	Asymptomatic; further paraesthesia (case 1); Pain, facial asymmetry.	Q1, Q2, Q3, Q4	Mixed-density pattern; poorly defined borders; unerupted teeth	Hyperdense image, hypodense halo; cortical resorption and bulging	Spindle-cell fibrocellular tissue; trabecular bone; multiple spheroidal cementum-like masses	Normal levels of AP, osteocalcin, T3, T4, TSH, and PTH	Biopsy; extraction; curettage; antibiotic therapy (case 1); follow-up (case 2)

F, female; M, male; NR, not reported; USA, United States of America; UK, United Kingdom; Q1, right upper quadrant; Q2, left upper quadrant; Q3, left lower quadrant; Q4, right lower quadrant; AP, alkaline phosphatase; T3, triiodothyronine; T4, thyroxine; TSH, thyroid-stimulating hormone; PTH, parathyroid hormone
